# DNA Methylation Dysfunction in Chronic Kidney Disease

**DOI:** 10.3390/genes11070811

**Published:** 2020-07-16

**Authors:** Diego Ingrosso, Alessandra F. Perna

**Affiliations:** 1 Department of Precision Medicine, School of Medicine, University of Campania “Luigi Vanvitelli”, 80138 Naples, Italy; 2 Department of Medical Translational Sciences, School of Medicine, University of Campania “Luigi Vanvitelli”, 80131 Naples, Italy; alessandra.perna@unicampania.it

**Keywords:** CKD, ESRD, dialysis, renal failure, DNA methylation, methylation reactions, epigenetic regulation, disease markers

## Abstract

Renal disease is the common denominator of a number of underlying disease conditions, whose prevalence has been dramatically increasing over the last two decades. Two aspects are particularly relevant to the subject of this review: (I) most cases are gathered under the umbrella of chronic kidney disease since they require—predictably for several lustrums—continuous clinical monitoring and treatment to slow down disease progression and prevent complications; (II) cardiovascular disease is a terrible burden in this population of patients, in that it claims many lives yearly, while only a scant minority reach the renal disease end stage. Why indeed a review on DNA methylation and renal disease? As we hope to convince you, the present evidence supports the role of the existence of various derangements of the epigenetic control of gene expression in renal disease, which hold the potential to improve our ability, in the future, to more effectively act toward disease progression, predict outcomes and offer novel therapeutic approaches.

## 1. Introduction

Kidney disease mostly refers to chronic kidney disease (CKD), which is a frequent complication of common metabolic, inflammatory and cardiovascular diseases. CKD represents the bulk of renal failure cases; these conditions evolve chronically in a matter of several years. On the other hand, various predisposing extra-renal (e.g., cardiovascular shock, thrombosis of the renal artery) or renal (e.g., toxic drug-induced tubular necrosis), or urinary (e.g., ureter obstruction) causes may rapidly (days or hours) evolve towards acute kidney injury (AKI), a condition which, if not promptly reversed, may soon require replacement therapy or evolve towards CKD. Various lines of evidence support the existence of epigenetic alterations in CKD, particularly as modifications of DNA methylation either global or at specific sites, or both. The meaning of these alterations, relevant to possible dysfunction of the epigenetic control of gene expression and the issue of their possible implication in the disease progression and the occurrence of CKD complications, are discussed, as well as the potential role of DNA methylation modification as disease markers. 

This review is organized in chapters, the first of which is dedicated to the epidemiology of renal disease, thus focusing on the global extent of the phenomenon and of its complications, which may as well relate to epigenetics. The subsequent chapters will be dedicated to specific aspects of renal disease and DNA methylation, which are relevant to: (I) anemia and erythropoietin in CKD (II) metabolic and biochemical aspects (e.g., hyperhomocysteinemia (HHcy), a strong cardiovascular risk factor with high prevalence in CKD. (III) Inflammation, fibrosis and disease progression. (IV) Epigenetic alterations of diagnostic, prognostic and/or therapeutic potential. (V) Onconephrology. 

## 2. Chronic Kidney Disease: A Worldwide Pandemic

**Figure 1:**
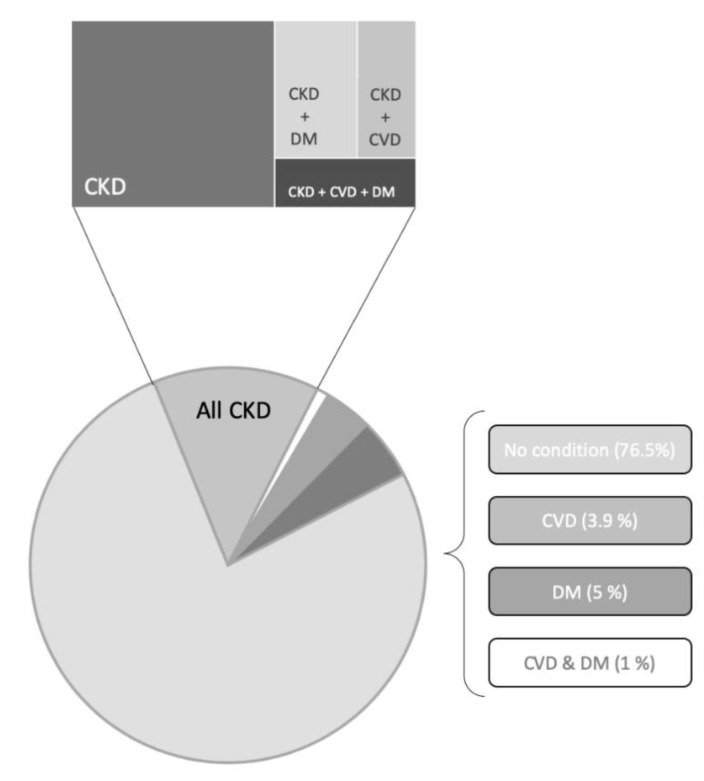
Prevalence of Chronic Kidney Disease (CKD) and other associated and underlying diseases in the United States of America. Data are from the National Institute of Diabetes and Digestive and Kidney Disease—U.S. Department of Health and Human Services [modified from: [5]. In the pie graph CKD accounts for 13.6 % of the total area. CKD; chronic kidney disease. DM; diabetes mellitus. CVD; cardiovascular disease.

Chronic kidney disease (CKD) is defined as abnormalities of kidney structure or function, present for more than 3 months, with implications for health, monitored by a reduced glomerular filtration rate (GFR) and/or other markers, such as proteinuria [1,2]. Kidney disease is steadily increasing worldwide, accounting for about 10% of the general population [3,4]. This is due to classical underlying causes, such as glomerulonephritis, as well as to a dramatic increase of a number of highly prevalent noncommunicable diseases, particularly hypertension and diabetes, whose frequent complication is kidney end-organ damage (Figure 1).

The endpoint in the clinical history of CKD patients is end-stage renal disease (ESRD), characterized by a GFR <15 mL/min/1.73 m^2^, the retention of at least 88 different solutes (so-called “uremic toxins”) and, eventually, the need for renal replacement therapy (dialysis or transplantation) [6]. This is a clinically-defined situation, see [2], affecting only about 1% of overall CKD patients, characterized by the uremic syndrome, i.e., the terminal clinical manifestation of kidney failure, frequently including an association of signs and symptoms related to the CKD complications listed in Table 1. Most of CKD patients are indeed affected by a high, premature death rate due to these complications, as discussed in the next paragraphs [2,3]. 

CKD is characterized by many frequent complications, which often threaten the patients’ lives (Table 1). Many, if not all, CKD complications have been associated with altered DNA methylation patters and/or deranged expression of epigenetically regulated genes by DNA methylation. In some cases, these findings suggest new potential therapeutic strategies. The availability of modern molecular biology nanobiotechnologies prompted substantial improvements in the study of DNA methylation in various diseases, thus including CKD, since the turn of this millennium, as described in the following sessions. In Table 1, all common complications in CKD are listed. Anemia and cardiovascular disease (CVD) are particularly important because anemia is almost constantly associated with CKD and requires careful intervention in order to restore hematocrit to an acceptable target value, without increasing blood viscosity and consequently further worsening CVD risk factors. On the other hand, CVD is the major cause of mortality in CKD. The implications of anemia and cardiovascular disease in CKD are indeed particularly important from an epidemiological and medical standpoint, and they also hold intriguing relationships with DNA methylation, as reported in the next two paragraphs. 

## 3. Anemia and Erythropoietin in CKD

Erythropoietin (EPO) alteration is the most typical and, certainly, the best detailed example of the potential involvement of derangements of DNA methylation in the pathogenesis of a common CKD complication: anemia. EPO is an essential erythroid growth factor, which is produced in the kidney by interstitial fibroblasts. Its production is modulated by local oxygen pressure and triggered by hypoxia. Early key work established that transactivator hypoxia-inducible factor-1 (HIF-1) modulates the expression of oxygen-regulated genes, including erythropoietin. This oxygen-dependent regulation is modulated by a CpG methylation of the DNA-binding site for HIF-1 [14]. In particular, it was initially shown that methylation levels of a CpG island-encompassing gene promoter and a 5’-untranslated region (5’-UTR) inversely correlated with erythropoietin expression. CpG methylation silences erythropoietin expression of the gene by both I) recruiting a methyl-CpG binding protein to the promoter, which is driven by high-density methylation at the 5’-UTR, and II) methylation of CpGs in the proximal promoter hampering the association with nuclear trans-acting factors [15]. DNA methylation inhibitors restore erythropoietin production in fibrotic murine kidneys; indeed, the DNA methyltransferase 1 (DNMT1) inhibitor 5-aza-2′-deoxycytidine (5-aza) is able to restore EPO production in primary mouse myofibroblast (MF-REP) cultures by decreasing DNA methylation in the EPO-gene promoter [16]. It has been also clarified that HIF actually consists of an α/β heterodimer which, during hypoxia, binds to hypoxia-response-elements (HREs) at the target gene loci [17]. Structural characterization and bioinformatics studies clarified that HIF-1α was the original HIF isoform from the EPO locus, while HIF-2α and HIF-3α were identified later on. HIF-1α and HIF-2α may play different roles in various anemia conditions; HIF-2α, for example, mediates nucleosome disassembly, in the mechanism of hypoxia-dependent erythropoietin induction. Animal studies have clarified that, when oxygen is available, HIF is inactivated by post biosynthetic modifications of basic amino acid residues in the α subunits [17], an oxygen-dependent function which is accomplished by proteins containing a HIF-prolyl hydroxylase domain (PHDs) [7]. Sato and coworkers recently showed that, in CKD related anemia, there is a disruption of the mechanism linking hypoxia with the consequent EPO induction. This is due to (I) metaplasia of renal EPO producing cells (REPs) into myofibroblasts (MF-REPs); (II) the pathological hyperactivation of PHDs as the result of kidney damage [7]. Although PHD inhibitors (e.g., roxaduxat) have been considered in the treatment of CKD-related anemia, their action may be partly neutralized, however, as CKD progression proceeds, since the HIF-2α and EPO encoding gene promoter regions are hypermethylated and therefore epigenetically silenced in MF-REPs, thus hampering the expression of these genes [7]. This is indeed a field of active research for personalized treatment applications in CKD patients, provided that a robust assessment of disease stage is accomplished. 

## 4. CKD and Cardiovascular Risk Increase: The Case for Hyperhomocysteinemia and Folate Intake, Relevant to DNA Methylation

CKD is affected by a very high cardiovascular mortality [18]. Directly after the turn of this millennium, Robert N. Foley wrote: “Without intervention, premature cardiovascular disease is virtually certain in progressive chronic kidney disease (CKD)”. [19]. Unfortunately, the situation has not improved much over the first lustrum of this century, despite extensive studies on both traditional and non-traditional cardiovascular risk factors, such as anemia and hyperhomocysteinemia, in CKD [8,20]. 

**Figure 2:**
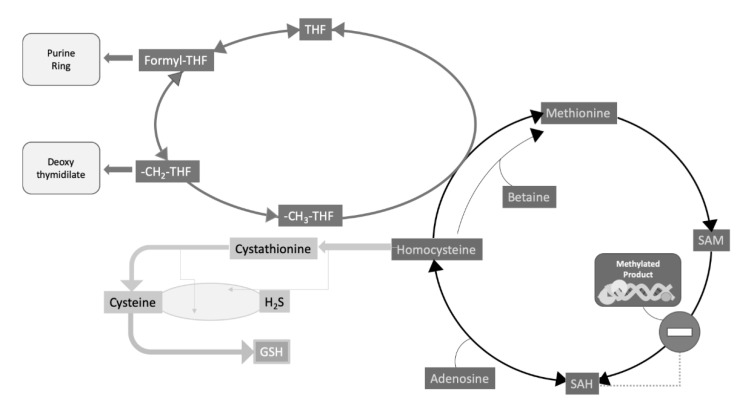
Homocysteine metabolism, folate cycle and methyl transfer reactions. Methionine is supplied by the diet; in that it is an essential amino acid. Homocysteine is generated from methionine through three subsequent reactions: (I) the expensive biosynthesis of *S*-adenosylmethionine (SAM) by the consumption of all phosphate bonds of ATP; (II) Methyl transfer from SAM to over fifty different acceptors, including small molecules and macromolecules (nucleic acids and proteins); (III) Hydrolysis of SAM demethylated product: *S*-adenosylhomocysteine (SAH) yielding adenosine and homocysteine. SAH is a powerful competitive inhibitor of SAM-dependent methyltransferases, therefore its enzymatic disposal is crucial to prevent its buildup within the cell thus creating the conditions for such inhibition to actually take place. (IV) homocysteine in turn can be re-methylated to methionine, though a couple of “rescue” reactions, dependent on either 5-methyltetrahydrofolate (5 -CH_3_-THF), as the major reaction also requiring cobalamine (vit. B12) as well, or betaine as a secondary reaction. Reactions, from reaction I to IV, define the methionine–homocysteine cycle. Alternatively, homocysteine can be transsulfurated to cysteine in a pyridoxal phosphate (vit. B6) -dependent pathway. One of the major products of further cysteine metabolism is the biosynthesis of glutathione (GSH) a major intracellular scavenger for reactive oxygen species (ROS). An alternative product of transsulfuration is hydrogen sulfide (H_2_S), the third gaseous vasodilator, after NO and CO. Interestingly enough, folates are involved in both DNA methylation, though the replenishment of the methionine–homocysteine cycle, and DNA biosynthesis, in that 5,10-methylenetetrahydrofolate (5,10 -CH_2_-THF) and formyl-THF are needed for the biosynthesis of important DNA nucleotide building blocks. In case of homocysteine remethylation, the CH_3_-THF cosubstrate for this methionine rescue pathway is generated by the catalytic activity of the enzyme methylenetetrahyfolate reductase (MTHFR; EC 1.5.1.20) (not shown).

Homocysteine lays at a typical intersection between one carbon (C_1_) metabolism, centered on the biochemical role of folates and folate cycle, and sulfur amino acid metabolism (Figure 2). Hyperhomocysteinemia, a pathological increase of this amino acid concentration in circulation, is a strong and independent cardiovascular risk factor and may be attributed to various causes (Table 2). 

Homocysteine connection to a higher risk of thrombosis was described in the second half of the last century; however, its involvement as a cardiovascular risk factor, based on observational studies, was particularly underscored in the 1990s [24]. In 1996, Robinson and coworkers published a paper which recognized a significantly higher prevalence of hyperhomocysteinemia in kidney failure patients and potentially identified two crucial vitamins involved in homocysteine metabolism, B6 and folate, as potential culprits [25]. Unfortunately, a number of intervention studies, using folate as a homocysteine-lowering measure, while being partially effective in decreasing homocysteine levels, did not show any substantial capability to reduce cardiovascular risk. It was put forward, however, that this could be the result of a number of confounding factors [20]. It should be pointed out, in this respect, that, in CKD, a derangement in folate receptor expression was reported to occur in CKD patients in hemodialysis, which may contribute to folate resistance, thus providing a partial explanation for the low folate effectiveness detected in some epidemiological studies [26]. On top of the controversies about homocysteine and folate intervention studies, a key report showed that folate was effective in significantly reducing cardiovascular mortality in a large cohort [27].

**Figure 3:**
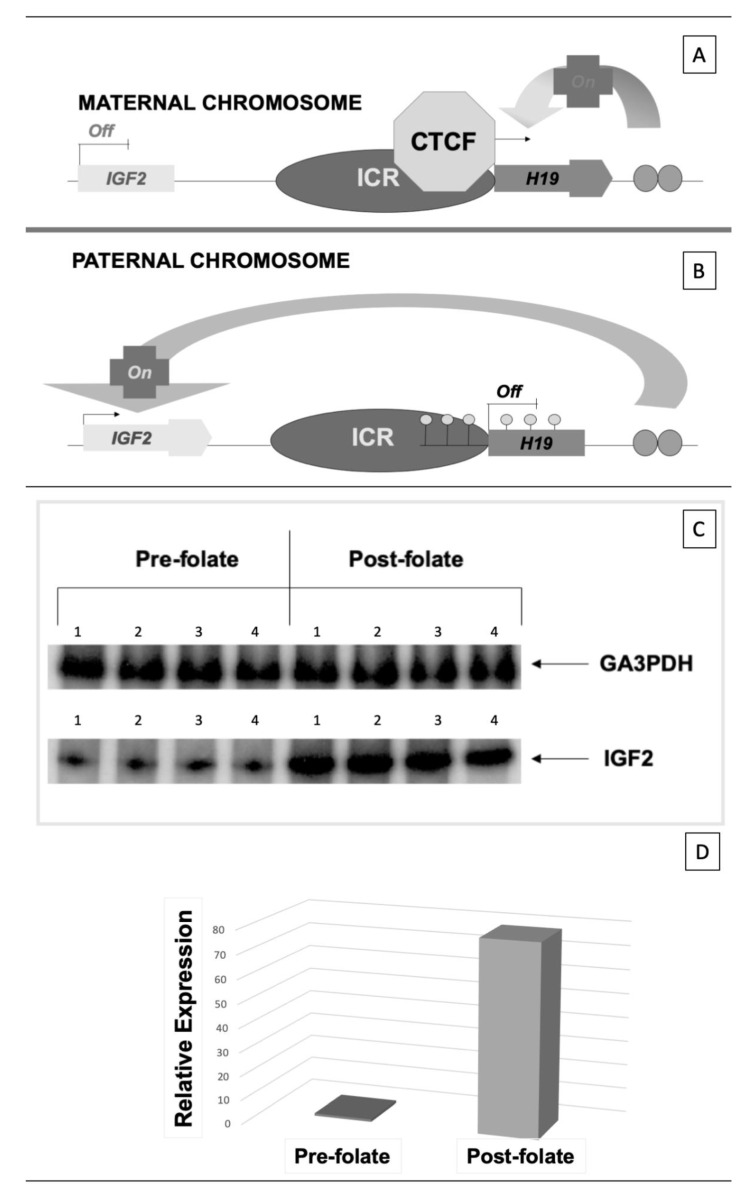
Effect of a folate treatment on the expression of the imprinted gene *IGF2*, in hyperhomocysteinemic uremic patients. (**A**,**B**); schematic representation of the inverse regulatory mechanisms for the selective monoallelic expression of *H19* and *IGF2* from the paternal chromosomes. The two genes are under the control of the same imprinting control region (ICR). Differential methylation is responsible for the differential expression of each imprinted gene according to parent-of-origin effects. On the maternal chromosome, absence of ICR methylation induces insulation of *IGF2*, through the interaction of the ICR with the trans factor CTCF, and allows monoallelic expression of *H19* (**A**). Methylation of the paternal ICR, ensures silencing of *H19*, allowing the permissive activity of a downstream enhancer on the *IGF2* promoter (**B**). Panel C–D; four CKD patients in end-stage renal disease, treated with renal replacement therapy (hemodialysis), were subject to folate wash out (prefolate), then a standard folate treatment (methyltetrahydrofolate) was administered (post-folate). Pre-folate and post-folate levels of expression of *IGF2* mRNA were measured before and after folate treatment in four CKD- end-stage renal disease (ESRD) patients. (**C**): semiquantitative analysis of labeled PCR products; (**D**): real time PCR; for details see [28].

Thus, given the extreme entangled metabolic roles of folate, it appears clear that it is, probably, very difficult to work out the various pieces of such a complicated puzzle. However, regarding the topic of the present article, perhaps one of the most striking impacts in the studies on homocysteine and CKD was the evidence that folate administration, in CKD patients in hemodialysis, had a significant effect on the epigenetic control of the expression of some imprinted genes [28]. In particular, it was found that: (I) patients’ DNA from peripheral mononuclear cells, resulted to be generally hypomethylated; it was hypothesized that the high SAH, found in hyperhomocysteinemic patients, may contribute to inhibit DNA methylation; (II) examining the expression of the imprinted gene couple *H19*/*IGF2*, it was found that *H19* was abnormally biallelically expressed, while *IGF2* was silenced; (III) after the administration of folate in these subjects, the normal monoallelic expression of both imprinted genes was restored along with a general increase of DNA methylation levels. An example of the above results is shown in Figure 3. 

As a whole, these results led to the general conclusion that a deficient folate status is able to influence the epigenetic control of gene expression by inducing an abnormal biallelic expression of imprinted genes, which under normal conditions are monoallelically expressed, by methylation-dependent silencing of one of the two parental alleles. These alterations are partly reversible upon folate supplementation. Remarkably, this may help in explaining why folate deficiency during early pregnancy is associated with a higher risk of neural tube defects possibly induced by a deficiency of the methyl group donor SAM (Figure 2), resulting in both elevated homocysteine and abnormal DNA methylation. 

More recently, it has been found that hyperhomocysteinemia may induce glomerular damage mediated by oxidative stress and DNA methylation impairment [29]. 

These results sustain the interpretation that acquired diseases, such as CKD, may be associated with significant changes in the epigenetic control of gene expression. On the other hand, modifications in the living microenvironment and/or the personal habits may trigger opposite changes in DNA methylation patterns, thus contributing to either the mechanisms of some diseases or to counteract some pathologic modifications. In the same context, additional evidence, brought forward by independent groups, spoke in favor of the involvement of methylation reactions and sulfur amino acid metabolism in CKD (Table 3). 

From a biochemical standpoint, it is well established that SAM is the universal methyl donor for almost fifty different methyltransferases [44,45] (Figure 2). All these enzymes share, indeed, some structural motifs, involved in methyl donor binding [45,46]. It is well known that macromolecule methylation is a feature pertaining to both DNA and proteins [47,48] with special regard to histones. Recently, histone methylation has become a major hotspot for research on both cancer [49] and vascular diseases [50], which are among the leading causes of mortality [51]. In this respect, it has been proposed that (I) methionine–homocysteine modulates the SAM/SAH ratio and therefore the rate of SAM-dependent methylation under pathological conditions and that (II) hypomethylation on frequently modified histones is involved in the deregulation mechanisms of autoimmune and metabolic disorders, as well as in cardiovascular disease. Since the methionine–homocysteine cycle occurs in the cytosol, the modulation of transmethylation reactions in the nucleus requires a transfer of SAM/SAH/homocysteine between the two subcellular compartments. In addition, homocysteine clearance appears to be essential for genetic protection [52]. 

Transmethylation reactions rely on the proper function of the methionine–homocysteine cycle (Figure 2), firstly because the latter includes the pathway for the biosynthesis and regeneration of SAM. This is indeed synthesized from methionine and ATP, and is the methyl donor for all methyltransferases, including DNA methyltransferases. Second, but not less importantly, the methionine–homocysteine cycle provides for a way to replenish methionine supply through the “rescue” of homocysteine remethylation reactions, which also contributes to prevent homocysteine build-up. The main reaction for homocysteine remethylation requires methyltetrahydrofolate as the methyl donor, and the cobalamin-dependent enzyme, methionine synthase (*N*5-methyltetrahydrofolate–homocysteine methyltransferase; EC, 2.1.1.13), the product of the *MTR* gene. This methionine synthase is the only mammalian enzyme that metabolizes CH_3_-THF to regenerate the C_1_ transporter, i.e., THF (Figure 2), and its deficiency is one of the possible causes of rare form of hyperhomocysteinemia associated with leukoencephalopathy and neurological symptoms [53]. 

Since the 1990s [34,54], CKD has been found to be associated with (I) a number of metabolic derangements of the methionine–homocysteine cycle thus including high levels of SAH and a reduction of SAM/SAH ratio; (II) hyperhomocysteinemia; (III) altered transmethylation reactions. 

To further underscore the metabolic interactions between folate and B12, recently, the question has arisen about whether B12 and/or folate status may, and/or their supplementation, slow down CKD progression. Li and coworkers demonstrated that folic acid treatment was associated with a dramatic reduction in the odds of renal disease progression in subjects bearing mild or moderate CKD in the group showing higher B12 levels [55].

Despite some contradictory results of previous studies, recent findings further support the notion of a beneficial effects of the combined association of folic acid plus hypotensive drugs (ACE inhibitors) in significantly reducing the risk of developing new-onset proteinuria in diabetic and hypertensive subjects compared to the hypotensive drugs alone [56]. 

Recent reports underscore the importance of nutritional perturbation of B vitamin status, and potential phenotypic effects through the interactions with mutant genotypes. Riboflavin was supplemented in homozygous subjects for the MTHFR C677T polymorphism, a highly prevalent condition, characterized by hyperhomocysteinemia, particularly in the presence of a poor folate status. Riboflavin, the FAD precursor, is the coenzyme of MTHFR (Figure 2). In these subjects, the response of one carbon metabolism to riboflavin supplementation induced some specific effects on one carbon metabolism, consisting in the increase in SAM and cystathionine increase, compared to CC wild type subjects, thus underscoring the possible implications for the increased risk of hypertension, present in the TT genotype population, which in turn represents a major modifiable risk factor for stroke and CVD [57]. In addition, an influence on altered DNA methylation, both at the global and specific DNA levels, has been reported to occur, upon riboflavin supplementation, in a subset of homozygous in adults, bearing the same *MTHFR* C677T polymorphism. More precisely, TT homozygous for the *MTHFR* C677T polymorphism was associated with global and site-specific DNA hypermethylation at various sites, compared to the wild type CC homozygous controls. Riboflavin induced significant decrease of hypermethylation at a specific CpG site [58]. 

**Figure 4:**
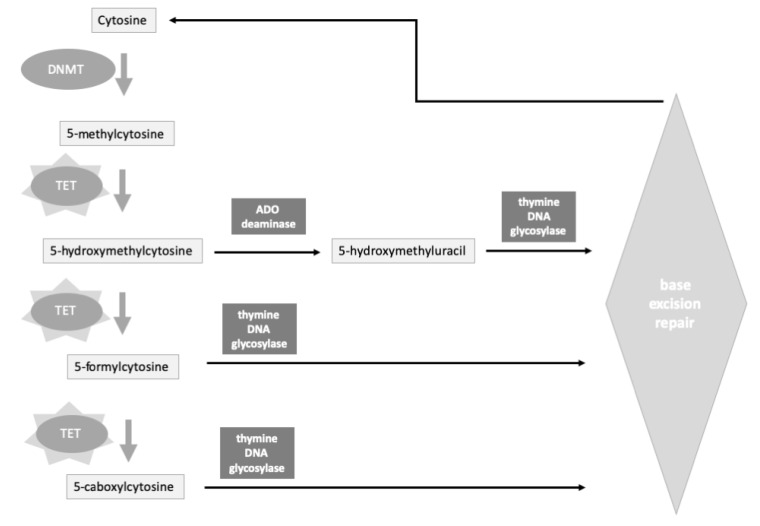
Schematic representation of the cytosine demethylation pathways and the role of the ten-eleven translocation (TET) enzymes.TET enzymes (star background) initiate the processing of 5-methylcitosine, by forming various derivatives, of the initial methylated cytosine precursor, in sequence (5-hydroxymethylcytosine, 5-formylcytosine, 5-carboxylcytosine). These are in turn further processed by the indicated enzyme systems, finally. See also [60,61] ADO, adenosine; DNMT, DNA methyltransferase; TET ten-eleven translocation.

It is worth highlighting the role of 5-hydroxymethylcytosine (5hmC) [59], a stable cytosine modifier endowed with its own functions in the enzymatic processing of 5-methylcytosine as a part of DNA demethylation system (Figure 4). 

5hmC is a rather stable product of DNA demethylation, together with 5-formylcytosine, and 5-carboxylcytosine, all generated by the catalytic action of the Ten-Eleven-Translocation (TET) family which oxidize methylated cytosines. At CpG-islands, 5hmC maintains promoters in the unmethylated state, whereas in intragenic sequences, 5hmC is suggested to have an inhibitory action on antisense transcription initiation [62]. Various pollutants have shown to increase the levels of DNA hydroxymethylation, such as nickel, cadmium, and arsenic which may alter DNA methylation patterns thus inducing epigenetic dysregulation of proto-oncogenes and oncosuppressors [63]. Arsenic may also act by depleting SAM and inducing reflections on DNA methylation levels. Arsenic exposure and 5hmC correlated positively in men and negatively in women, pointing to the existence of gender-specific differences. In addition, the presence of HHcy, as an indicator of poor B vitamin (B_12_ and folate) status, also represented, together with gender, a modifier of the association between arsenate and 5hmC [64].

## 5. DNA Methylation as a Potential Marker of Kidney Function, Inflammation and Fibrosis and CKD Progression

Kidney failure, internationally defined as end-stage renal disease (ESRD) or, less frequently, end-stage kidney disease (ESKD) can be part of the natural evolution of the disease, characterized by several complications. Clinical guidelines recommend the use of conventional disease markers for CKD consisting of measurements of glomerular filtration rate (GFR) or its indirect evaluation (eGFR) calculated from serum creatinine, by taking into account some anthropometric variables, including age, gender and ethnicity. Proteinuria is also recommended as a key criterion, according to clinical guidelines for CKD evaluation, classification, stratification and evaluation of disease progression. These clinical biomarkers may offer limitations, particularly in the elderly or in children, in the early CKD stages, with extreme body mass index values. Moreover, the use of serum creatinine may induce a deviation of eGFR results from the ideal behavior. Alternatively, eGFR estimation may be influenced by the presence of inflammation, steroid treatment and thyroid dysfunction, when using serum cystatin C instead of creatinine. Hence, a useful combination of creatinine and cystatin C has been proposed for eGFR evaluation [65]. 

A number of epidemiological factors connected with an increasingly aging population together with a growing incidence of the metabolic syndrome, characterized by the triad of hypertension, obesity and diabetes, underpin the high prevalence of CKD (Figure 1). From a pathophysiological standpoint, CKD is characterized by persistent inflammatory noxae, leading to fibrosis and loss of renal function (compromised GFR and proteinuria), eventually leading to ESRD and the need for replacement therapy. On the other hand, while TGF-β is the main regulator which drives fibrosis [66], epigenetic mechanisms are also a promising candidate, in this respect [67]. In addition, although new cures have been introduced in the last two decades, CKD treatment still offers limited therapeutic options, to block disease progression, thus drawing attention to the search for innovative approaches [68]. 

In the present paragraph, a number of studies were reviewed, which gave an outstanding contribution to the understanding of: (i) whether or not epigenetic modifications may influence individual susceptibility to develop CKD and the relevant mechanism involved; (ii) how various environmental factors may interact with the epigenome thus contributing to the occurrence of renal damage and dysfunction. 

At the beginning of this decade, there was a substantial change in the way the problem of “if and how” epigenetic modifications, associated with CKD, could be of pathophysiologic relevance. Apart from the widespread introduction of genome-wide techniques to address these questions, the search moved away from the analysis of global methylation as well as of well-known methylation-dependent gene models (e.g., imprinted genes), and became more focused on the identification of specific markers of disease progression related to the main morphological signatures of evolving CKD: local inflammation and fibrosis monitored by decaying GFR and proteinuria. Perhaps the first comprehensive analyses of genome-wide epigenetic alterations in CKD were performed by Zawada and coworkers [69] using SuperTAG methylation-specific digital karyotyping. Various candidate genes associated with proatherogenic and inflammatory processes were identified. Ten clinically CKD-stable and stable patients in hemodialysis were analyzed and compared with age- and sex-matched healthy controls. Over four-thousand significantly differentially methylated loci were identified in hemodialysis patients with respect to the control. Bioinformatic analysis allowed the identification of 52 potentially CVD-associated genes and 97 genes possibly relevant to infection or immune-associated disease conditions. Candidate genes were referred to various proatherogenic processes, thus including inflammation, angiogenesis, regulation of cell proliferation, lipid metabolism [69].

Later on, in 2017, an epigenome-wide analysis was published by Chu Ay et al. [70], which was accomplished on samples obtained from two large cohorts: 2264 whole blood specimens were from the ARIC Study and 2595 samples from the Framingham Heart participants, respectively. Analysis of epigenome-wide tags was associated with signs of altered eGFR, indicating the possible occurrence of CKD, in order to identify potential epigenetic signatures of renal dysfunction. The association with morphological alterations, often preceding and/or accompanying CKD, was also considered. Data allowed for the identification of nineteen CpG sites whose differential methylation resulted in being highly significantly associated with clinical or laboratory evidence of renal disease. In addition, biopsies from CKD patients were also analyzed and five CpG sites displayed consistent DNA methylation alterations, at the kidney cortex level, which could also be associated with the extent of renal fibrosis in the same samples. In particular, in these kidney cortex samples, methylation of leading CpGs was found associated with decreased renal *PTPN6* expression, higher eGFR, and a lesser degree of renal fibrosis. *PTPN6* product is a protein tyrosine phosphatase (PTP) family member. PTPs are indeed part of signaling pathways, regulating a number of cellular processes such as cell growth and differentiation, as well as mitosis and oncogenic transformation. PTPN6 protein product is primarily expressed in hematopoietic cells, where it acts as a key signaling regulator of multiple pathways. The N-terminus of PTP N6 contains two tandem Src homolog (SH2) phospho-tyrosine binding domains, which mediate its interactions with its phosphorylated protein substrates. These findings indicate that epigenetic variation at specific CpG sites may serve as signatures of associated kidney functional and morphological alterations of potential prognostic value. 

Klotho (EC 3.2.1.31) is an enzyme, encoded by the *KL* gene (HGNC:6344), belonging to type-I membrane proteins and related to β-glucosidases, and mainly expressed in the kidney and parathyroid glands. The triad, composed by α-Klotho, fibroblast growth factor-23, and its receptor, is involved in the pathogenesis of the mineral and bone disorder of CKD [71]. α-Klotho is subject to proteolytic ectodomain shedding from membrane catalyzed by ADAM17, a disintegrin and metalloproteinase, regulating the sorting between two forms: membrane Klotho (working as the FGF23 coreceptor) and secreted (soluble) Klotho, involved in ion channel/transporter and growth factor signaling pathways. As its very name suggests, Klotho is an anti-aging and anti-fibrotic protein and its early decline after renal injury is associated with aberrant DNA methylation [72]. These authors reported that TGFβ enhanced DNA methyltransferase 1 (DNMT 1) and DNA methyltransferase 3a (DNMT 3a) through the inhibition of miR-152 and miR-30a in both renal cells and mouse fibrotic kidneys, thus determining Klotho promoter hypermethylation. Consequently, they propose that aberrant TGFβ, miR-152/30a, DNMT1/3a and loss of Klotho form an epigenetic renal fibrotic cascade [72].

CKD is a frequent complication of diabetes. Gluck and coworkers studied DNA-methylation in samples from diabetic patients with CKD compared to non-CKD diabetic subjects. The authors identified a set of 65 genome-wide probes, whose methylation levels were significantly associated with the occurrence of renal fibrosis [73]. In the same work, the analysis of a total of 471 methylation probes was found consistent with the model that—taking into account DNA methylation—may significantly improve the prediction of renal function decline in this CKD patient subset. Epigenetic alterations are a candidate underlying mechanism to help explain how the chronic metabolic derangements, associated with diabetes, may contribute to the risk of renal disease onset in these subjects [73]. Fibrosis is indeed a key feature of kidney involvement in diabetes; in this respect, evidence indicated that, in diabetic nephropathy, TGF-β1 plays an important role as a trigger factor of the epithelial–mesenchymal transition and then in the pathogenesis of fibrosis. It has been found that epigenetic histone modifications may exert a significant effect in modulating upon TGF-β1 and some other downstream profibrotic genes [74]. 

Moreover, recent studies have rather focused on genome-wide linkage studies and the identification of epigenetic risk factors [75]. These associations have provided insights into disease pathophysiology, severity, and prognosis.

In conclusion, it should be pointed out that, at present, DNA methylation is, at most, a prospective biomarker of CKD. This is the reason why, in this section, the term “potential” is indeed absolutely necessary. At present, neither the international guidelines recommend the use of DNA methylation to judge CKD stage or progression, nor have laboratory methods been developed in order to do so, on a routine basis, in Laboratory Medicine. Additionally, no marker-guided therapy can presently be hypothesized based on DNA-methylation data. 

In this section, some potential applications of DNA methylation evaluation were described. However, some limitations do exist at present, which hamper the application to medical laboratory routine. These consist of sample and method standardization, relatively high costs of the relevant determinations, the intrinsic individual variability of DNA methylation levels, the presence of confounders (e.g., inflammation), and the possible effects of metabolic and/or environmental factors, possibly influencing the epigenetic layout.

## 6. DNA Methylation as a Potential Therapeutic Target and to Prevent CKD Progression

According to the notion that CKD is a multifactorial disease, we should expect that genetic and environmental factors interact in the disease pathophysiology. As a matter of fact, various genomic biomarkers for CKD have been described in the last decade [76], including *UMOD*, *SHROOM3* and *ELMO1. UMOD* encodes for a uromodulin also referred to as Tamm–Horsfall glycoprotein, a GPI-anchored glycoprotein, which is also the most abundant protein in normal urine [77]. Uromodulin is synthesized by kidney and localizes in cells lining the ascending limb of Henle and distal convoluted tubule and, together with uropontin, and nephrocalcin, it is indeed related with the formation of calcium-containing kidney stones [78]. Uromodulin mutations have been implicated in various renal pathologies. *SHROOM* family member 3 (SHROOM3) [79] is highly expressed in the liver, followed by the kidney, ovary, cerebellum, and spinal cord and its function is related to regulation of cytoarchitecture, particularly to a redistribution of microtubule regulator γ-tubulin. *ELMO1* (Engulfment And Cell Motility 1) [80] is involved in the regulation of cell motility and cytoskeletal rearrangements in phagocytosis and apoptosis. Some genetic alterations have been indeed associated with various kidney disease conditions or with their direct signs, including a decreased GFR or an increased circulating creatinine concentration. 

Two aspects have been considered: (A) the first is relevant to the potential role of gene alterations in the underlying kidney disease mechanisms. In this view, employing epigenetic and transcriptomic approaches may help investigate renal disease alterations, also identifying their related processes. (B) On the other hand, the potential practical identification of diagnostic and prognostic (epigenetic and transcriptomic) biomarkers may be useful in the attempt of combining the latter with classical disease markers in order to provide a more complete description of kidney diseases. 

Epigenetic modifications which may play a pathophysiological role in kidney disease, and which have been associated with renal structural and/or functional damage, include DNA methylation, histone modifications, and changes in microRNA levels. These epigenetic modifications are recognized as reversible to some extent and, therefore, represent a potential therapeutic target [67]. 

One key problem in the management of kidney disease is the prevention and monitoring of disease progression, which usually increases between stage 3a and 3b, i.e., when GFR falls below 60 mL/min/1.73 m^2^ and 45 mL/min/1.73 m^2^, respectively. 5-methyl-2′-deoxycytidine (5MedC) has been proposed as a DNA methylation marker. Onishi et al. studied the extent of 5MedC urinary excretion in over 300 CKD patients, in association with a number of other urinary markers, including albuminuria and α1-microglobulin (α1MG), together with other conventional CKD biomarkers. Using multiple logistic regression models, urinary 5MedC was significantly associated with the prediction of later CKD stages, and, in particular, predicted a 30% decline in the eGFR or a development of ESRD when combined with macroalbuminuria or an increased urinary α1MG excretion. The mechanism of such 5MedC alterations, as well as of their possible functional meaning, remains elusive at present, although it is worth noting the potential innovation of using the competitive enzyme-linked immunosorbent assay (ELISA), a widely employed laboratory medicine technique, for the measurements of urinary 5MedC [81]. 

Dysfunctions of DNA methylation have been related to renal fibrosis, a crucial final pathophysiologic step in the progression to ESRD [73], leading to the hypothesis that demethylating drugs may play a role in this respect [82]. Acute kidney injury (AKI) represents, in this regard, a particularly challenging condition, in that it often occurs insidiously, and common markers of kidney function in CKD do not become altered early during AKI onset. In consideration of the intriguing relationship between inflammatory response and epigenetic modifications, evidence is available that AKI, particularly during AKI-to-CKD transition—a frequently occurring situation—is associated with the activation of epigenetic regulatory mechanisms. This accompanies various clinical pathophysiological subsets, such as postrenal ureteral obstruction, prerenal ischaemia-reperfusion injury, tubular nephrotoxicity, as well as glomerulonephritis and polycystic kidney disease or diabetes [83]. On the other hand, other laboratory markers, e.g., kidney injury molecule-1 (KIM-1), neutrophil gelatinase-associated lipocalin (NGAL) KIM-1 or NGAL, have not proved adequate to provide solid prognostic information on renal function in a heterogeneous CKD population [84]. Based on this notion, encouraging results have been obtained from practical approaches aimed at counteracting the effects of a preclinical kidney injury. This has been attempted, for example, by the administration of compounds which induce a decrease of DNA methylation by either activating demethylation (e.g., hydralazine) or by inhibiting DNA methylation (e.g., 5-azacytidine and decitabine). Alternative approaches have been attempted by: (I) decreasing histone methylation (e.g., inhibiting histone methyltransferases); (II) increasing histone acetylation by inhibiting histone deacetylases (HDACs), by means of valproic acid, vorinostat, entinostat, increasing histone crotonylation (crotonate); (III) interfering with histone modification readers, e.g., inhibition of bromodomain and extra-terminal proteins (BETs) [83]. BETs constitute a protein family whose members interact with acetylated lysine residues on the histone tails, thus inhibiting interactions with acetylated histone (H3/H4) and recruiting chromatin-regulating proteins on the promoter region to regulate gene expression and repression, thus participating in the molecular mechanisms of various diseases and indeed representing potential therapeutic targets [85].

A frequent burden in CKD is a personal history of metabolic syndrome, in which many patients are chronically medicalized. On the other hand, oxidative stress is a common feature in this patient population. In a study published in 2017, the authors found that CKD patients in stages 3-4 displayed increased oxidative stress markers associated with whole-blood global DNA hypomethylation, measured by using capillary zone electrophoresis [86]. In the same patients, cholesterol-lowering treatments were associated with a dose-dependent restoration of both oxidative stress markers and DNA methylation levels, compared to the control, possibly mediated by the former. 

Conclusions from this section support the notion that present promising results may allow for the availability, in the future, of DNA methylation marker-targeted therapeutic approaches. 

## 7. Onconephrology: A New Frontier in Medicine

A discussion on epigenetics and kidney disease cannot be exhaustive without briefly mentioning the growing relevance of Onconephrology, a relatively young branch of medicine which originated from a merge of both Oncology and Nephrology [87]. Apart from the well-established notion that CKD patients display a higher cancer risk, partly related to immunosuppressive therapies for kidney transplantation, but also due to the effects of uremic toxicity, the field of Onconephrology regards many other issues, encompassing both the case of patients bearing cancer of the kidney, but also those who are affected by the secondary involvement of the kidneys in other malignancies and/or because of complications due to cancer therapies. The majority of kidney cancers are associated with mutations in the von Hippel–Lindau gene [88] and a small proportion are associated with infrequent mutations in other well characterized tumor-suppressor genes. More specifically, mutations of *PBRM1*, *SETD2*, *BAP1*, *KDM5C*, *KDM6A*, *MLL2* and relevant alterations of histone post-biosynthetic modifications, involved in chromatin organization and repair, have been reported to occur in renal carcinoma [89]. In addition, a number of other epigenetic alterations have been found to be associated with renal cancer, which include all major epigenetic mechanisms which are related to major chromatin and nucleosome remodeling, including DNA hypermethylation-dependent silencing at specific gene levels, histone modifications driven by mutations of histone modifying genes, microRNA dysfunctional alterations [90].

Renal cell carcinoma (RCC) is the most common type of kidney cancer (80% total neoplasia, circa). According to the new 2016 WHO classification, the major subtypes are clear cell RCC (65–70%), papillary RCC (15–20%) and the chromophobe RCC (5–7%) [91]. Differently from other cancers, in renal carcinoma cells, the search and findings of epigenetic alterations are even more predominant than the extent of somatic mutations found at individual gene levels. Among the alterations found, there are altered DNA methylation, and microRNA levels, as well as histone modifications, which have been detected in the main important signaling pathways in RCC, including von Hippel–Lindau disease tumour suppressor (VHL)-hypoxia-inducible factor (HIF) pathway, the WNT-β-catenin pathway, and various pathways involved in epithelial–mesenchymal transition. This is consistent with the interpretation that understanding the mechanisms of epigenetic reprogramming in these diseases will shed new light on the availability of new potential biomarkers and relevant therapeutic strategies [92]. A retrospective microRNA-related analysis was performed on formalin-fixed paraffin-embedded RCC, compared to normal kidney specimens. The results detected the potential miR-21-5p and miR-210-3p signature, as a putative biomarker for RCC clinical monitoring [93]. An interesting approach consisted in the systematic analysis of chromatin marks associated with Long non-coding RNA expression, in 475 primary clear cell RCC from the Cancer Genome Atlas (TCGA) [94]. Four distinct lncRNA subclasses could be associated with distinct clinicopathological and genomic features. Cluster C2 (23.4%) characterized the most aggressive tumors, with the most severe staging and grading as well as the worst survival [95]. 

It should be pointed out that such epigenetic signatures in RCC are not yet routinely used for diagnostic, prognostic and therapeutic targeting, at present. However, because of their involvement in important regulatory pathways, such epigenetic alterations are considered with potential implication as biomarkers for early disease detection. In addition, since they are potentially reversible, it is reasonable to expect that they may become, in the future, useful markers for effective response to treatments. 

As mentioned, CKD patients have an increased risk of cancer, with cancer of the renal tract and thyroid particularly increased [96]. There is also a graded association between the severity of CKD and cancer mortality. Transplant patients have an increased risk of cancers associated with immune deficiency and with virus infection, including genitourinary sites, Kaposi sarcoma, lymphoma, melanoma, and cancers of the head and neck. The presence of proteinuria also increases the risk. If epigenetic modifications induced by uremic toxicity may influence cancer risk is yet to be proven with adequately powered studies. 

In conclusion of this section, from a pathophysiological standpoint, a remarkable aspect of Onconephrology consists of the dual-faceted relationship between cancer and CKD. On one hand, a number of factors (e.g., immunosuppressive therapies, other drugs) increases the risk of cancer in CKD patients. On the other hand, an intrinsically increased risk of CKD is present in some cancer patients, because of either the localization of the neoplasm (e.g., renal cancer), or because of the potential renal toxicity of some anticancer drugs (e.g., cisplatin and derivatives, and new biological agents).

## 8. Synopsis and Conclusive Remarks

Kidney disease is a worldwide growing medical emergency. Renal failure, both occurring as chronic kidney disease (CKD) or initiating as an acute kidney injury (AKI) evolving towards chronicization is characterized by a number of complications, including chronic anemia, mineral and bone dysfunction and significantly increased cardiovascular disease risk. There is, therefore, a need to identify additional non-invasive biomarkers that may be useful in clinical practice to help improve CKD diagnosis and prognosis, through a correct evaluation of disease progression, and, possibly, guiding therapeutic management. 

Epigenetic mechanisms regulate important processes involved in the regulation of gene function and of downstream cellular responses. Several reports identified associations between CKD and epigenetic regulation, with special regard to DNA methylation. Then, the influence of frequent variables in the CKD patient population has been substantially underscored by differential results from independent groups. Studies reached conclusions which strongly support notions that are somewhat in contrast with traditional definition of epigenetics, as a process including all heritable changes in gene expression, occurring during mitosis and meiosis, although not encoded in the DNA sequence itself. In fact, CKD is an example those acquired disease conditions, and/or related behavioral habits or therapeutic interventions may indeed influence the epigenome structure and function. In this respect, new models may become available for the study of epigenetic modifications, their potential pathophysiological and translational meaning in the conditioning of the mechanisms for kidney disease progression and search for relevant biomarkers to be transferred to clinical practice.

However, at present, limitations do exist. As stated in the relevant section and in Table 1, anemia is perhaps the most direct example of the involvement of DNA methylation in CKD, because of its participation in EPO regulation. The possible role of DNA methylation in participating in the pathogenesis of CKD-associated anemia was discussed. The involvement of DNA methylation in all the other complications of CKD is, at most, an intriguing working hypothesis, not a definitive conclusion. In order to support the routine use of epigenetic parameters, particularly as DNA methylation measurements, for the clinical management of kidney disease patients, two aspects need to be refined: (I) an extensive re-evaluation of the limits of applicability of the results from experimental studies; (II) a robust improvement and automation of lab methods, in order to be applied, at a reasonable low cost, to routine diagnostics. 

## Figures and Tables

**Table 1:** CKD complications.

Complication	Related Lab Markers	Parameters/Symptoms/ Nosological Entities	Prevention/Therapy	Ref.
Anemia	Low Hb conc Low Ht	Fatigue Dyspnoea Hypoxia	Erythropoietin	[7]
Cardiovascular disease (CVD)	Dyslipidemia Hyperhomocysteinemia Other metabolic alterations	CVD events (myocardial infarction, stroke, thrombosis)	Control of cardiovascular risk factors	[8]
Electrolyte imbalance	Hyperkalemia Hyponatriemia (*)	Heart dysfunction (ECG abnormalities, arrhythmias)	Specific electrolyte correction (cation exchange resins; diuretics)	[9]
Fluid retention	Increased plasma volume; Decreased plasma oncotic pressure from protein loss	Edema	Diuretics (furosemide, tolvaptan) Dialysis (end-stage renal disease)	[10]
Gout	High uric acid (**)	Arthritis Kidney stones CVD risk factor	Xanthine oxidase inhibitors (allopurinol, febuxostat)	[11]
Metabolic acidosis	Arterial Blood Gas (ABG) alterations (low pH, low bicarbonate, low BE, compensatory low paCO_2_)	Organ injury (headache, chest and bone pain, palpitation, dyspnoea, nausea, vomiting, weakness, and bone pain. anxiety, mental derangements, seizures, coma, heart arrhythmia.	Specific therapy based on correction of pH and bicarbonate alterations.	[12]
Mineral and Bone Disease	High PTH Hyperphosphatemia Low vitamin D3 (1,25-dihydroxy-colecalciferol)	Secondary hyperparathyroidism Pathological fractures Metastatic calcifications	Phosphate binders (Sevelamer; aluminum hydroxide); Calcium sensing receptor agonists 1,25-dihydroxy-colecalciferol (calcitriol)	[13]

paCO_2_; CO_2_ arterial partial pressure. BE; base excess. (*) Hyponatremia can be a side effect of some diuretics. (**) High uric acid can be both the consequence of CKD and being responsible for kidney stone and, eventually, CKD.

**Table 2:** Principal causes of hyperhomocysteinemia (HHcy) and associated disease conditions.

Group	Disease/Condition/Compound	Pathophysiology	Symptoms/Laboratory Findings
Genetic			
	Homocystinuria	CBS mutations/deletions	CVD/ectopia lentis/Severe HHcy ^(a)^
		Methionine synthase	CVD/Severe HHcy; low Met
		Other ^(b)^	Severe HHcy (low Met)
	Polymorphysms	MTHFR C677T ^(c)^	(CVD)/ Moderate/Intermediate HHcy (incostant/conditioned) ^(d; e)^
		MTHFR A1298C	(CVD)/ Moderate/Intermediate HHcy (incostant/conditioned) ^(d; e)^
Nutritional deficiency			
	Folate	Met-Hcy cycle deficiency	High Hcy (moderate)
	B12	Met-Hcy cycle deficiency	High Hcy (moderate); methylmalonic aciduria; low HoloTC; check also for gastric intrinsic factor (GIF) deficiency
	B6	Transsulfuration deficiency	Drug interaction; alcohol abuse
Drug administration			
	Antifolates	Methotrexate ^(f)^	Cancer or inflammatory disease (Psoriasis/arthritis)
	Anti-parkinsonian drugs	L-DOPA ^(g)^	Parkinson disease
Acquired			
	CKD	Uremic toxins? ^(h)^	see also Table 1

^(a)^ Severe HHcy>101 mmol/L [n.v. 10 (f)-12 (m) mmol/L]. ^(b)^ Methionine synthase reductase deficiency [21]. ^(c)^ HHcy when associated with a poor folate status. ^(d)^ HHcy; Moderate 16–30 mmol/L; Intermediate 31–100 mmol/L. ^(e)^ CVD when ^(b)^ is present; neural tube defects in the absence of folate supplementation/fortification. ^(f)^ Synergistic with MTHFR C677T. ^(g)^ Mechanism likely due to catecholamine load (COMT substrates) [22]. ^(h)^ See [23].

**Table 3:** Milestones in the history of disease-relevant derangements of one carbon metabolism, sulfur amino acid metabolism, and transmethylation reactions.

Finding(s)	Reference(s)
Ex vivo protein methylation inhibition and decreased intracellular SAM/SAH concentration ratio in CKD	[30]
Impaired DNA methylation and vascular endothelial cell growth induced by homocysteine, in vitro	[31]
In vivo studies on transmethylation inhibition in CKD	[32,33]
Raised blood SAH concentration in CKD patients ex vivo	[34,35]
Defective macromolecule methylation and repair associated with growth factors activation, lipid deposition and increased vascular smooth cell proliferation in atherogenesis, ex vivo studies	[36,37]
In vivo global DNA hypomethylation in MTHFR C677/ polymorphism (mononuclear blood cells)	[38]
In vivo global DNA hypomethylation and response to folate (mononuclear blood cells)	[28]
Metabolic effects on DNA methylation. A very exhaustive review article	[39]
Mice lacking MTHFR develop severe steatosis and have elevated plasma homocysteine, increased hepatic content of SAH and reduced SAM	[40]
HHcy is associated with higher SAH in liver and brain and induces tissue-specific changes in H19 methylation and expression in *CBS* knockout mice	[41]
Severe HHcy affects methylation potential in the renal tissue and lowers erythropoietin expression following CO induced intoxication in rats	[42]
Suppression of Klotho expression by protein-bound uremic toxins is associated with increased DNA methyltransferase expression and DNA hypermethylation (in vitro)	[43]
